# The expansion of the Foundation Programme in psychiatry^†^

**DOI:** 10.1192/pb.bp.115.052126

**Published:** 2016-08

**Authors:** Jennifer Perry, Ann Boyle, Simon Wessely

**Affiliations:** 1 South London and Maudsley NHS Foundation Trust; 2 Leicestershire Partnership NHS Trust; 3 King's College London

## Abstract

The *Broadening the Foundation Programme* report has led to an expansion in the number of psychiatry foundation placements. This change will have far-reaching benefits for foundation doctors doing psychiatry, no matter what their future career intentions. Doctors will develop a better understanding of mental illness, they will improve their communication skills and they will gain experience of working within multidisciplinary teams. Recruitment into psychiatry is also likely to improve. The Royal College of Psychiatrists is putting in place a number of measures to ensure that placements are of a high quality so that foundation doctors have a good experience of psychiatry.

Our society is changing; increasing numbers of people are living for longer and with more complex and chronic conditions. Although the importance of mental health in the context of health, illness and well-being is not new, what is new is the increasing acceptance of this and the recognition that there is indeed ‘No health without mental health’.^1^ Whether we call it ‘patient-centred’, ‘whole-person’, ‘holistic’ or by any other name, people are now demanding a healthcare system that integrates the physical, psychological and social spheres, as recognised in the priorities of the recent *Five Year Forward View* proposed by NHS England^2^ and endorsed across the healthcare spectrum. Doctors' education, embedded in the Foundation Programme, must follow these developments.

## Changes to the Foundation Programme

One tangible result of these transformations has been a substantial change in the foundation years, the obligatory 2 years that all doctors must undertake before moving into specialty training. Within their foundation year 1 (FY1) and foundation year 2 (FY2) posts, doctors undertake a number of placements, usually lasting 3–4 months each. From August 2015, the targets set are that at least 80% of foundation doctors should undertake a community or integrated placement and from August 2017 this will rise to 100%. Psychiatry is counted as a community placement.^3^ The target set by the psychiatry taskforce is to have 22.5% of all FY1 and 22.5% of all FY2 placements in England in psychiatry.^3^ This means that nearly half of all doctors will participate in at least 4 months of postgraduate psychiatry training, which represents a significant increase.

The purpose of these changes is to ensure that future doctors have more transferable skills such as communication and empathic understanding. They will develop the ability to work successfully in multidisciplinary teams and will acquire a better understanding and appreciation of the importance of mental health, irrespective of their subsequent career pathway. Exposure to psychiatry will also help to challenge the stigma of mental disorder.^4^ Finally, it may encourage recruitment into the specialty. Health Education England recognises that there are problems with recruitment into psychiatry and it is working with the Royal College of Psychiatrists to improve this.^5^

The Royal College of Psychiatrists recognises the importance of these changes, and has made the expansion of the foundation year programme one of its key priorities.

### Numbers

Table 1 shows the most recent figures from the UK Foundation Programme Office (UKFPO). Currently, 10.4% of all FY1 and 13.4% of all FY2 placements are in psychiatry and it should be noted that there is variation between the UK countries: England has the highest proportion of FY1 placements (20.9%), with Scotland, Wales and Northern Ireland having very few. The figures show that there is still some way to go to achieve the psychiatry taskforce targets.

**Table 1:** Proportion of foundation placements in psychiatry in the UK

	Placements in psychiatry,^a^ %	
	FY1	FY2
England	20.9	11.8

Scotland	4.6	20.1

Wales	5.3	13.1

Northern Ireland	0.0	31.2

UK total	10.4	13.4

FY, foundation year.

a. Source: The UK Foundation Programme Office, www.foundationprogramme.nhs.uk.

### Quality of placements

Health Education England have mandated that the targets set out in the *Broadening the Foundation Programme* report^3^ are achieved. However, it is one thing to ensure the fulfilment of a target, as this is merely a means to an end, not the end in itself. It is essential that these new placements are of high quality if they are to deliver on the goals previously stated. Put simply, unless foundation doctors have good professional and learning experiences during their placements, the results may be at best neutral, and at worst counterproductive. So we must ensure not just the number, but also the quality, of the new placements.

### Impact on recruitment

It is well recognised that there have been serious problems in recruitment to specialty training in psychiatry, nevertheless some improvements have recently been noted. In its report, the Centre for Workforce Intelligence indicates that recruitment into psychiatry training in England at the core trainee (CT1) level is improving, with fill rates increasing from 87 to 97% between 2009 and 2013.^6^ However, at higher specialty training fill rates have fallen from 82 to 80% between 2013 and 2014. Old age psychiatry has the lowest fill rates, which is of concern given the ageing population and the actual and predicted demand. There are also regional variations: south west and north east regions of England had the lowest number of specialty trainee (ST4) doctors in training per capita. The report highlights the risk of the future consultant psychiatrist workforce supply being unable to meet the predicted increase in patient demand.

A paper by Goldacre *et al*^7^ stresses that the experience of psychiatry as a student and in early medical training is important in determining career choice. A study by Kelley *et al*^8^ found that there was a significant association between a foundation psychiatry placement and appointment to core psychiatric training.Their paper describes how, proportionally, significantly more doctors with a Foundation Programme placement in psychiatry went into psychiatry training compared with foundation doctors with no psychiatry placement. We can look more widely at the literature to examine what might attract foundation doctors into psychiatry. Brockington & Mumford^9^ look at recruitment and discuss factors which encourage medical students into psychiatry, which include the opportunity to undertake a holistic approach to caring for patients, the opportunity to get to know patients in-depth, the breadth of the field and its interactions with other disciplines.^10^ Dein *et al*^11^ found that the most important factors for consultant psychiatrists in their choice of career were empathy for patients, working conditions and experience of the subject as a student. These factors could also play a role in the recruitment of foundation doctors.

Old age psychiatry placements are particularly suitable for foundation doctors as they provide a wide view of services and address the key issue of integrating training across the health and social care system. This enables doctors to acquire important transferrable competencies. Foundation placements in old age psychiatry also provide an opportunity to introduce doctors to a specialty which is currently experiencing significant recruitment difficulties.

## What is the College doing?

The Royal College of Psychiatrists is putting in place a number of measures to support the expansion of psychiatry posts in the Foundation Programme. The College offers free associate membership to foundation doctors, which enables them to access journals and learning resources. There are College-sponsored events, newsletters, regional and national prizes and bursaries which are aimed specifically at foundation doctors. The College has recently produced national guidance on the quality control and quality management of posts^12^ to help ensure they are of a high standard. Educational resources are being created to support trainers in supervising foundation doctors. There will be a focus on the development of academic psychiatry foundation posts and on setting up a national network for psychiatry foundation leads.

## Opportunities and risks

We cannot overemphasise that the purpose of the expansion of the foundation years is not to recruit more psychiatrists; if that happens it would be a welcome, and indeed likely, side-effect. Trainers need to recognise that most of the new foundation doctors will not opt for a career in psychiatry. Nonetheless, this is a unique opportunity to create a future generation of doctors with greater mental health literacy. Given the fact that most of the people with mental disorders are not treated by secondary mental health services, this may have a greater impact on the mental health of the nation than improvement in specialty recruitment.

We must therefore openly acknowledge some of the opposition to this programme that may come from outside but also from within our own ranks. We know that other doctors may have preconceived negative views about foundation psychiatry placements, which may echo those of the doctors in training.^3^ It is pleasing and reassuring to note that preliminary evidence is suggesting that these negative views are starting to begin to change. Feedback from recent College meetings with foundation doctors who have completed psychiatry placements has been remarkably positive. Many of these doctors reported that they enjoyed their placements, in particular the 1 h per week protected time for supervision with their consultant. However, many described how multidisciplinary teams continue to treat them like medical students, seeing them as observers rather than doctors in their own right. Foundation doctors are at the beginning of their professional careers and teams need to recognise this.

Many doctors voiced concerns about professional and social isolation and losing contact with their colleagues in the acute hospitals; this is something which programme directors need to be mindful of. It can, in part, be tackled by foundation schools ensuring foundation doctors in psychiatry are grouped together, or by developing posts in specialties such as liaison psychiatry where doctors still remain in the acute hospital. Some mental health trusts have developed mental health teaching programmes and Balint groups specifically for foundation doctors, which provide further opportunities for peer support.

A lack of exposure to acute care or procedural experience relevant to the curriculum was also raised at the meetings. To counter this, local education and training boards can consider designing posts where psychiatry foundation doctors spend 1 day a week in the acute trust or doing medical on-call work. Opportunities to acquire core medical skills within their day-to-day clinical work (e.g. undertaking physical health assessments of newly admitted in-patients) should also be available to foundation doctors and care should be taken to ensure this is well supervised.

In summary, the expansion of the Foundation Programme in psychiatry is an opportunity to give doctors a positive and valuable experience of working in the specialty. Preliminary evidence relating to foundation doctors' experience of psychiatry posts is positive. The College has a responsibility to ensure that posts are of a good quality, as the literature shows that experiences of psychiatry in early medical training are important in determining career choice.^6^ If posts are not of a high quality, then this will simply reinforce existing prejudices and diminish, not increase, the chances that junior doctors will add psychiatry to their list of possible career options. Enhancing the foundation doctors' experience of psychiatry will be one of the Royal College of Psychiatrists' biggest challenges, but if done well, it potentially has far-reaching benefits.
